# A Nanozymatic-Mediated Smartphone Colorimetric Sensing Platform for the Detection of Dimethyl Phthalate (DMP) and Dibutyl Phthalate (DBP)

**DOI:** 10.3390/bios13100919

**Published:** 2023-10-08

**Authors:** Wenhui Li, Xuecheng Zhang, Haojie Zhang, Cheng Zhang, Yingjie Chen, Cong Li, Yonghong Hu, Xiaoping Yu, Biao Zhang, Xiaodong Lin

**Affiliations:** 1 College of Food and Light Industry, Nanjing Tech University, Nanjing 211816, China; li3781692@njut.edu.cn (W.L.); hyh@njut.edu.cn (Y.H.); 2 Zhejiang Provincial Key Laboratory of Biometrology and Inspection & Quarantine, College of Life Sciences, China Jiliang University, Hangzhou 310018, China; zhangxuecheng@cjlu.edu.cn (X.Z.); zhj20@cjlu.edu.cn (H.Z.); zhangcheng@cjlu.edu.cn (C.Z.); cyj221@cjlu.edu.cn (Y.C.); yxp@cjlu.edu.cn (X.Y.); zb@cjlu.edu.cn (B.Z.); 3 Agriculture and Rural Bureau of Zhuozhou, Zhuozhou 072750, China; lic1995@126.com; 4 Zhuhai UM Science & Technology Research Institute, Zhuhai 519000, China

**Keywords:** colorimetric immunoassay, dimethyl phthalate (DMP), dibutyl phthalate (DBP), Pt@Au nanozyme, two-component simultaneous detection

## Abstract

Plasticizers are a type of toxic substance that may remain in food, posing significant health risks including carcinogenic, teratogenic, mutagenic, and other adverse effects. In this study, a novel strategy was employed by combining Pt@Au nanozymes with high catalytic properties to created two catalytic signal probes, designated as Pt@Au@Ab1 and Pt@Au@Ab2, specifically designed for the detection of dimethyl phthalate (DMP) and dibutyl phthalate (DBP). These catalytic signal probes served as the foundation for the development of a colorimetric immunoassay, enabling the simultaneous detection of both DMP and DBP. The colorimetric immunoassay is capable of detecting DMP in the range of 0.5–100 μg/L with a limit of detection as low as 0.1 μg/L and DBP in the range of 1–32 μg/L with a low limit of detection of 0.5 μg/L. The developed immunoassay can be used for the determination of the DMP and DBP in baijiu and plastic bottled drinks. The recovery rate is in the range of 96.4% and 100.5% and the coefficient of variation is between 1.0% and 7.2%. This innovative colorimetric immunoassay offers a robust tool for the simultaneous quantification of DMP and DBP in real samples.

## 1. Introduction

Food safety has always been a major issue concerning people’s livelihoods [1,2,3]. Dimethyl phthalate (DMP) is a plasticizer, mainly found in glass products [4,5,6]. Plasticizer as the main additive in plastic products can increase product plasticity and improve product strength, it can also play a softening role, and it is widely used in toys, food packaging materials, medical blood bags and hose, vinyl flooring and wallpaper, cleaning agents, lubricants, personal care products (such as nail polish, hair spray, soap, and shampoo), and hundreds of other products [7,8,9,10]. Dibutyl phthalate (DBP) is the most commonly used plasticizer of polyvinyl chloride, which can make products exhibit good softness and it is the main plasticizer in food packaging materials [11,12]. These compounds have been shown to be harmful to the human reproductive system, due to reproductive toxicity, embryonic toxicity, and genotoxicity [13,14]. Therefore, it is of great significance to carry out the detection of plasticizers.

In addition to illegal food additives, the source of plasticizers in food also includes environmental pollution and infiltration of food packaging materials. Spraying coatings, incineration of plastic waste, volatilization of plasticizers in agricultural films, etc., make phthalates (PAEs) enter the natural environment, migrate, transform, accumulate in environmental media such as the soil, water, and atmosphere, and they can enter the biological body, accumulate in natural foods such as poultry, seafood, vegetables, and fruits, thereby causing great harm [15]. In addition, in terms of food packaging materials, there is no chemical covalent bond between PAEs and the plastic matrix, so it will dissolve when it comes into contact with the oils contained in the packaged food and pollute the food. Beverage wine, especially Chinese liquor, is a good solvent for phthalate plasticizers because of its high alcohol content [16]. In the process of liquor production, transportation, and storage, causing contact with plastic products, the phthalates contained in them will migrate to the wine, and the migration amount is related to the contact time, thus increasing the residual number of plasticizers in the wine, causing food pollution and bringing risks to human health [17]. In order to prevent and avoid the harm of plasticizers to the human body, it is of great significance to research and develop a method for rapid detection of plasticizer content [18].

At present, the main detection methods of plasticizers include thin-layer chromatography [19], gas chromatography [20] and gas chromatography-mass spectrometry [21], high-performance liquid chromatography (HPLC) [22], high-performance liquid chromatography-tandem mass spectrometry (HPLC-MS/MS) [23]. The above detection methods have the characteristics of high detection accuracy and good reproducibility, but the samples entering the instrument require complex preprocessing, such as extraction and purification, and require certain professional operators, which has certain limitations. Therefore, simple sample pretreatment, an easy-to-operate detection process, and a detection method with high sensitivity are needed to be developed for the rapid detection of plasticizers.

Nanozymes are nanomaterials that mimic the activity of natural enzymes [24,25,26]. It has the specificity of natural enzymes, and the properties of nanozymes are more stable and the application range is wider. Among them, noble metal nanozymes have the advantages of high and adjustable catalytic activity, good biocompatibility, and easy surface modification, etc., [27,28,29]. Currently, it is widely used in the detection of foodborne pathogens, biotoxins, pesticide residues, veterinary drug residues, environmental pollutants, and cancer markers [30,31]. Noble metal nanozymes combined with colorimetric detection methods have high sensitivity for the analysis of target objects. The analysis speed is fast. In this study, a Pt@Au nanozyme with high catalytic performance was prepared, and two catalytic signal probes were prepared by combining the antibodies of DMP and DBP. A colorimetric immunoassay for the simultaneous detection of DMP and DBP was established based on the traditional competitive enzyme-linked immunosorbent assay (ELISA).

## 2. Materials and Methods

### 2.1. Reagents and Instruments

Dimethyl phthalate (DMP), dibutyl phthalate (DBP), diethyl phthalate (DEP), dipropyl phthalate (DPRP), di-N-myl phthalate (DPP), di-N-hexyl phthalate (DNHP), dicyclohexyl phthalate (DCHP), dinonyl phthalate (DNP), di-N-octyl phthalate (DNOP), di-(2-ethyl) hexyl phthalate (DEHP), diphenyl phthalate (DPHP), diisobutyl phthalate (DIBP), diisononyl phthalate (DINP), butyl benzyl phthalate (BBP), bis(4-methyl-2-amyl) phthalate (DMPP), ethyl phthalate (2-methoxy) phthalate (DMEP), 1,2-benzenedicarboxylic acid, bis(2-ethoxyethyl) ester (DEEP), bis(2-N-butoxyethyl) phthalate (DBEP), disodium hydrogen phosphate dodecahydrate, sodium dihydrogen phosphate dodecahydrate, degreasing milk powder were purchased from Aladdin Biochemical Technology Co., Ltd. (Shanghai, China). Ovalbumin (OVA), glutaraldehyde, 3, 3′, 5, 5′-tetramethylbenzidine (TMB), N, N-dimethylformamide (DMF), Tween-20, K_2_PtCl_4_, HAuCl_4_, Pluronic F127 were purchased from Sigma-Aldrich (Shanghai, China). The antibodies of DMP and DBP were purchased from Kejie Industrial Development Co., Ltd. (Shenzhen, China). Other chemical reagents are analytical grade. Varioskan LUX multimode microplate reader was obtained from Thermo Fisher Scientific Co. (Waltham, MA, USA). Ultrapure water was prepared using a Millipore pure water meter (Millipore, Burlington, MA, USA). The accuracy of the detection results of the developed method was verified by Waters HPLC System with 2998 PDA Detector (Shanghai, China).

### 2.2. Preparation of Coating Antigen

The coating antigen of DMP and DBP was prepared by using the glutaraldehyde method. The main experimental process is as follows. Firstly, 10 mg of OVA was dissolved in a glass bottle with 1.0 mL PBS solution (pH 7.4). Then, 2.78 mg of DMP (or DBP) was dissolved into 100 μL DMF, and ultrasonic treatment was performed. In the stirring state, the above solution was slowly added into the OVA solution several times and thoroughly mixed. Then, 10.0 μL 25% glutaraldehyde was added to the above reaction system and stirred at room temperature overnight. The excess glutaraldehyde was removed by PBS dialysis for 72 h. The coating antigen of DMP and DBP was stored at 4 °C for later use.

### 2.3. Preparation of Signal Probes and Coated Microporous Plate

The detailed process for the preparation of the material is listed in the support material [32]. The catalytic signal probes of DMP and DBP (Pt@Au@Ab1, Pt@Au@Ab2) were prepared as follows. Firstly, 3.0 mL of the aforementioned Pt@Au nanomaterial (diluted 10 times) was added into two centrifuge tubes, respectively. The pH of the Pt@Au nanomaterial was adjusted to 8.8 by 0.2 M K_2_CO_3_, and 20 μg of anti-DMP antibody and 40 μg of anti-DBP antibody were added, respectively. After a reaction for 1 h, 30 μL of PBS (pH 7.4, containing 10% BSA) was added and incubated for 30 min to block the site of the Pt@Au. The free antibodies or proteins were removed by centrifugation with 10,000 r/min for 20 min. Finally, the Pt@Au@Ab1 and Pt@Au@Ab2 catalytic signal probes were redissolved and stored at 4 °C for later use.

Ninety-six microporous plates were divided into DMP and DBP standard liquid areas and detection areas according to the support materials shown in Figure S1. The micropores standard liquid areas and detection areas of DMP were coated with DMP-coating antigen (0.05 μg/well). The standard liquid region and the micropores in the detection region of DBP were modified by the DBP-coating antigen (0.05 μg/well), and then the whole microplate was sealed with 200 μL 3 mg/mL defatted milk powder to prevent experimental errors caused by non-specific adsorption.

### 2.4. Test Process

DMP and DBP standards with different concentrations of 50 μL were added to the unit micropores in the DMP detection area and the DBP detection area, respectively, and the concentration gradients for DMP and DBP were formulated as 0.5, 1.0, 5.0, 10, 50, 100 μg/L and 1.0, 2.0, 4.0, 8.0, 16.0, 32.0 μg/L, respectively. Then, the catalytic signal probes of the DMP and DBP signal probes were added and incubated for 20 min for competitive reaction. The reaction solution in the micropores was removed, and each well was washed with 250 μL PBS buffer (0.01 M, pH 7.4) 2 times. And 200 μL of 0.5 mM TMB color-development solution was added for 2 min. A colorimetric immunoassay for simultaneous detection of DMP and DBP was constructed according to the B value obtained by the software reading RGB in the smartphone and the concentrations of DMP (or DBP).

### 2.5. Samples Pretreatment and HPLC Verification

The samples pretreatment of baijiu and plastic bottled drinks were directly diluted 10 times for the developed colorimetric immunoassay. Sample pretreatment for HPLC verification was conducted by referring to GB 5009.271-2016 with some modifications. First, 5 mL of baijiu and plastic bottled drinks samples were taken into test tubes, followed by the addition of 3 mL n-hexane, and the mixture solution was shaken for 3 min. The supernatant was transferred to a clean tube after being centrifuged at 5000 r/min for 2 min, and 3 mL n-hexane was added into the precipitate and extracted again. The above supernatant was combined together and blown to 2 mL under nitrogen at 40 °C for purification.

The acetone and n-hexane were successively added into the SPE purification column to activate functional groups on the inner surface of the column. The effluent was discarded, and the extracted sample was added at a flow rate controlled within 1 mL/min. A total of 5 mL acetonitrile was added to the elution target molecule, the effluent was collected, and then dried with nitrogen at 40 °C. Then, 1 mL of acetonitrile was added for subsequent instrumental analysis. The detection conditions of high-performance liquid chromatography (HPLC) are as follows. The detection wavelength was 230 nm. The flow rate of the mobile phase was set under the following conditions: 0 min, 50% acetonitrile; 10 min, 90% acetonitrile; 15 min, 100% acetonitrile; 20 min, 50% acetonitrile.

## 3. Results

### 3.1. Detection Principle of the Developed Colorimetric Immunoassay

**Scheme 1:**
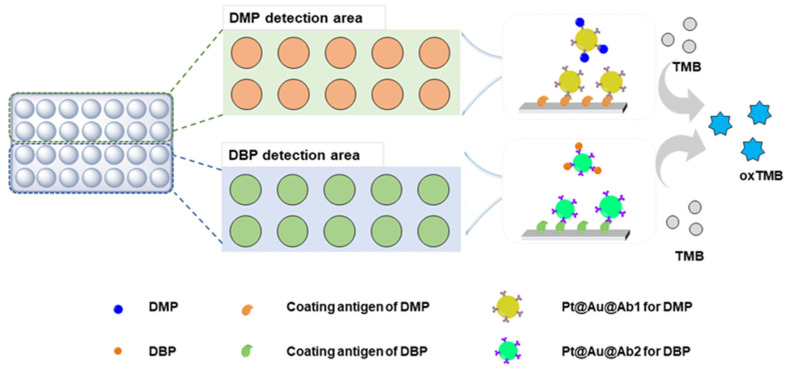
Detection principle of the developed colorimetric immunoassay.

The detection schematic diagram of the constructed colorimetric immunoassay is shown in Scheme 1. The design of the standard liquid test area and sample test area diagram (Figure S1) are listed in the Supplementary Materials. Firstly, encapsulated antigens (DMP and DBP) and DMP and DBP as targets (antigens) can bind competitively to Pt@Au@Ab1 and Pt@Au@Ab2, respectively. Then, as the concentration of the target in-creases, more of the probe (Pt@Au@Ab1 and Pt@Au@Ab2) is bound and washed away by the buffer, which in turn results in fewer nanoprobes binding to the encapsulated antigen. Subsequent to the addition of the TMB chromogenic substrate, the increase in the con-centration of the present target decreases the oxidized TMB, and a gradual decrease in blue color can be noticed. Subsequently, the color was recorded using the color picker APP in the smartphone, and a standard curve was built based on the relationship between the blue channel (B value) and the concentration of the target to achieve the detection of DMP and DBP in the samples.

### 3.2. Characterization of Pt@Au

**Figure 1:**
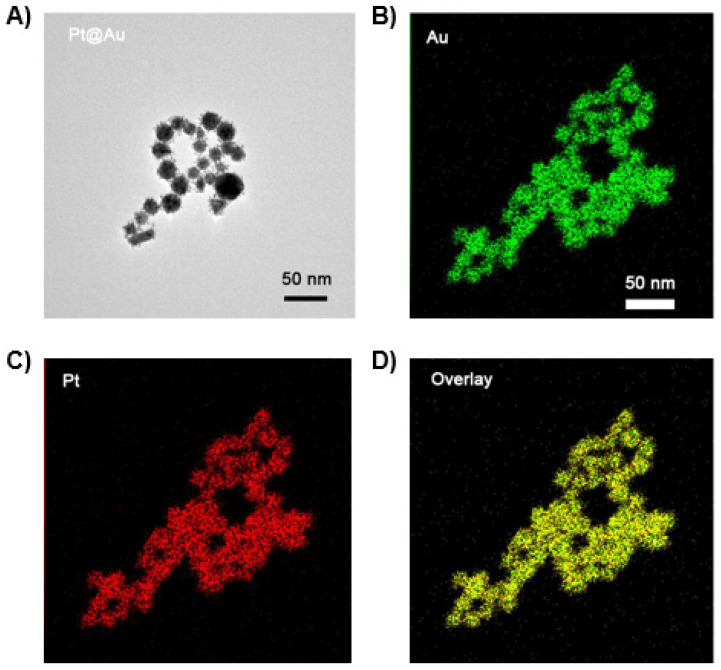
Characterization of Pt@Au nanozyme. TEM image of prepared Pt@Au nanomaterials (**A**) and the distributions of Au (**B**), Pt (**C**), and merged (**D**).

The Pt@Au samples were diluted 20 times and prepared in the laboratory, and the morphology was analyzed by a Japan JEOL JEM-2800EX microscope operated at 200 kV (Tokyo, Japan). The results are shown in Figure 1A. The synthesized Pt@Au nanozyme has a uniform particle size distribution. The ImageJ software was used for particle size statistics, and the statistical analysis of the particle size of Pt@Au nanozyme was about 18.52 nm (Figure S2). As shown in Figure 1B,C, the distribution of Au and Pt elements was uniform; an overlay of Pt@Au nanozyme is shown in Figure 1D. To four centrifuge tubes containing color-developing solution, 10 μL ultrapure water was successively added, along with 5 μL, 10 μL, and 20 μL of the diluted Pt@Au nanozyme solution. The B value of the color-developing solution was obtained. The results showed that the Pt@Au nanozyme had the ability to catalyze the complex color-developing substrate (Table S1). Therefore, subsequent visual detection experiments of DMP and DBP could be carried out.

### 3.3. Optimization of Detection Conditions

**Figure 2:**
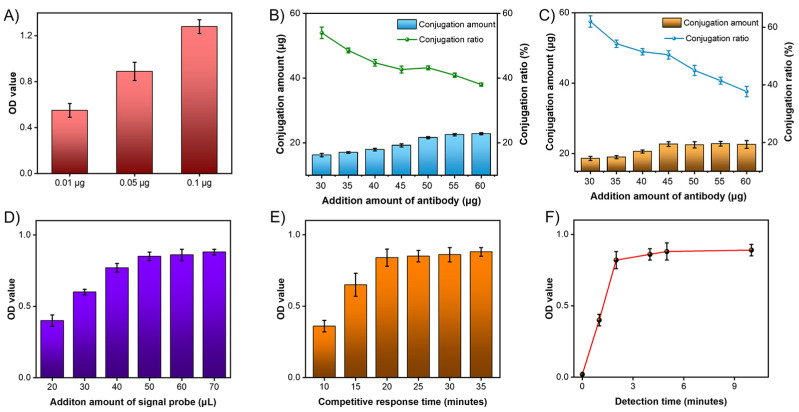
Optimization of detection conditions. (**A**) The added amount of coating antigen of DMP and DBP, (**B**,**C**) the added amount of antibody of DMP and DBP for probe preparation, (**D**) the added amount of catalytic signal probes, (**E**) the competitive reaction time, and (**F**) the catalytic reaction time.

In order to improve the sensitivity and stability of the colorimetric sensing method for the detection of DMP and DBP, the added amount of coating antigen of DMP and DBP, the added amount of antibody for probe preparation, the added amount of catalytic signal probes, the competitive reaction time, and the catalytic reaction time were optimized. The coated amount of each microwell was set to 0.01 μg, 0.05 μg, and 0.1 μg, respectively. In Figure 2A, when the coated amount is 0.01 μg, the OD_450_ value read is less than 0.8; when the coated amount is 0.05 μg, the OD_450_ value read is 0.89; when the coated amount is 0.1 μg, the OD_450_ value read is greater than 1.2. The added amount of 0.05 μg per well was selected for subsequent experiments. During the preparation of DMP and DBP signal probes, the added amount of antibody was 30, 35, 40, 45, 50, 55, and 60 μg. The antibody concentrations of supernatant were detected by the BCA kit to calculate the coupled antibody on the surface of the Pt@Au nanozyme. When anti-DMP and anti-DBP antibodies were 50 μg and 45 μg, respectively (Figure 2B,C), the coupling contents were 21.6 μg and 22.7 μg, and the coupling rates were 43.2% and 50.4%, respectively. Anti-DMP and anti-DBP antibody additions of 50 μg and 45 μg were used to prepare the catalytic signal probes of DMP and DBP, respectively. The addition amount of catalytic signal probes was set at 20, 30, 40, 50, 60, and 70 μL, according to the OD_450_ value, and 50 μL of the addition amount of catalytic signal probes was selected (Figure 2D). The competitive response time (10, 15, 20, 25, 30, 35 min) was optimized, and 20 min was selected for the follow-up experiment (Figure 2E). In Figure 2F, the final color development time is 2 min.

### 3.4. Development of Standard Curve for DMP and DBP 

**Figure 3:**
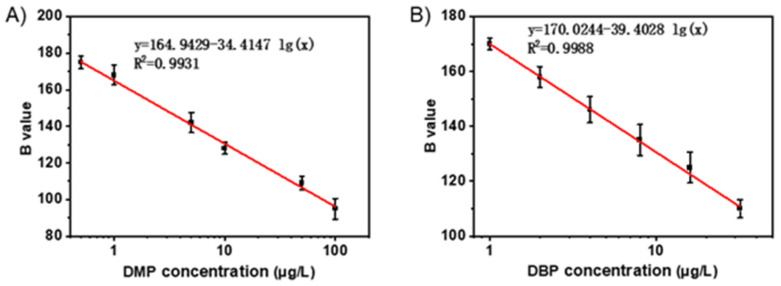
**(A**) Standard curve for DMP and (**B**) DBP in the constructed colorimetric immunoassay.

Under the optimal detection conditions, a sensitive colorimetric immunoassay was constructed to detect both DMP and DBP in a PBS solution. As shown in Figure 3A, the detection standard curve equation of DMP is y = 164.9429 − 34.4147 lg(x) (R^2^ = 0.9931) and the detection range is 0.5–100 μg/L with a limit of detection (LOD) of 0.1 μg/L, which was calculated based on 3σ. As shown in Figure 3B, the standard curve equation for the detection of DBP is y = 170.0244 − 39.4028 lg(x) (R^2^ = 0.9988), the detection range is 1–32 μg/L with an LOD of 0.5 μg/L. The same antibody was used to construct an ELISA for DMP and DBP, and the calculated detection limits (IC_15_) for DMP and DBP were 2.95 μg/L and 10.20 μg/L. Compared with the ELISA of DMP and DBP (Figures S3 and S4), the constructed colorimetric immunoassay has higher sensitivity, and the detection limit of the constructed immunoassay was increased by about 20–30 times.

### 3.5. Specific Evaluation of the Constructed Colorimetric Immunoassay

The specificity of the constructed colorimetric immunoassay is evaluated by detecting structural analogues and congeners, such as diethyl phthalate (DEP), dipropyl phthalate (DPRP), di-N-myl phthalate (DPP), di-N-hexyl phthalate (DNHP), dicyclohexyl phthalate (DCHP), dinonyl phthalate (DNP), di-N-octyl phthalate (DNOP), di-(2-ethyl) hexyl phthalate (DEHP), diphenyl phthalate (DPHP), diisobutyl phthalate (DIBP), diisononyl phthalate (DINP), butyl benzyl phthalate (BBP), bis(4-methyl-2-amyl) phthalate (DMPP), ethyl phthalate (2-methoxy) phthalate (DMEP), 1,2-benzenedicarboxylic acid, bis(2-ethoxyethyl) ester (DEEP), bis(2-N-butoxyethyl) phthalate (DBEP). As shown in Table 1, cross-reactivity of the above-mentioned substances for the constructed colorimetric immunoassay are all less than 5%, and this is mainly due to the high specificity of the anti-DMP antibody, which also indicates that the developed colorimetric immunoassay has good specificity for DMP. In the specific evaluation of the constructed colorimetric immunoassay for DBP, the cross-reactivity of BBP, DIBP, DNP, and DMP are between 20% and 40%, and the cross-reactivity of others are less than 10%. The structural formulae of DNP and DBP are similar, which may be the reason for the relatively high cross-reactivity. However, the molecular structure of BBP, DIBP, and DMP are not close to the molecular structure formula of DBP, but they have a high cross-reactivity, which may be caused by the low purity of the starting substance DBP and the presence of BBP, DIBP, and DMP impurities in the preparation process of commercial antibodies. This indicates that when there is a certain concentration of BBP or DIBP or DNP or DMP in the sample, the constructed colorimetric immunoassay has a certain degree of false positive probability for the detection of DBP. In summary, the proposed colorimetric immunoassay can be applied to the simultaneous detection of DMP and DBP.

### 3.6. Analytical Application in Real Samples

The stability and applicability of the constructed colorimetric immunoassay were evaluated by the detection of DMP and DBP in real samples. In this study, baijiu and plastic bottled drinks were selected as actual samples for the detection of DMP and DBP background concentrations. As shown in Table 2, the background concentrations of DMP and DBP were not detected in the baijiu and plastic bottled drinks samples. Next, the samples were added at levels of 20 μg/L and 50 μg/L of DMP and DBP, respectively. The constructed colorimetric immunoassay was used to detect the concentrations of DMP and DBP in the samples with DMP and DBP added, and the detection results were confirmed by ELISA. As shown in Table 2, there was no significant difference (*p* > 0.05) through statistical analysis between the detected results of the colorimetric immunoassay and those of the ELISA, and the detected data of correlation coefficient was greater than 0.99.

Meanwhile, the recovery rates of DMP and DBP in the samples detected by this colorimetric immunoassay ranged from 96.4% to 100.5%. The coefficient of variation (CV) of this colorimetric immunoassay was between 1.0% and 7.2%. The recovery of ELISA was between 98.2% and 102.0%, and the coefficient of variation was between 1.0% and 5.6%, indicating that the colorimetric immunoassay has good stability, and it can be used for the simultaneous detection of DMP and DBP in actual samples.

## 4. Discussion

The Pt@Au nanozyme with good catalytic properties was prepared, and two kinds of catalytic signal probes (Pt@Au@Ab1, Pt@Au@Ab2) were prepared by modifying anti-DMP and anti-DBP antibodies on the surface of the Pt@Au nanozyme. Based on the catalytic signal probes, a highly sensitive and rapid colorimetric immunoassay for the simultaneous detection of DMP and DBP was constructed. The detection ranges of DMP and DBP were 0.5–100 μg/L and 1–32 μg/L, respectively, and the LODs were 0.1 μg/L and 0.5 μg/L, respectively. Compared with the traditional ELISA with the same antibodies and coating antigen, this colorimetric immunoassay effectively improves the detection sensitivity and saves the detection time by 50–70 min. The proposed colorimetric immunoassay has been successfully applied to the simultaneous detection of DMP and DBP in baijiu and plastic bottled drinks, and it is expected to provide a tool for the detection of DMP and DBP.

## Figures and Tables

**Table 1:** Specific evaluation of the proposed colorimetric immunoassay.

Name	Structural Formula	Cross-Reactivity for DMP (%)	Cross-Reactivity for DBP (%)
DMP	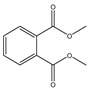	100	20.5
DBP	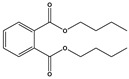	<5	100
DEP	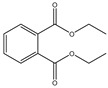	<5	<10
DPRP	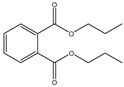	<5	<10
DPP	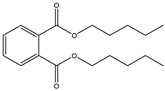	<5	<10
DNHP	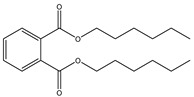	<5	<10
DCHP	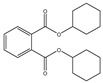	<5	<10
DNP	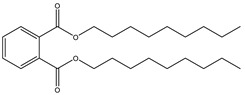	<5	40
DNOP	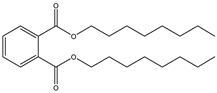	<5	<10
DEHP	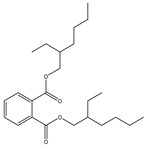	<5	<10
DPHP	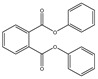	<5	<10
DIBP	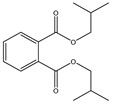	<5	21.2
DINP	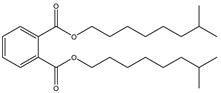	<5	<10
BBP	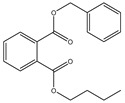	<5	20
DMPP	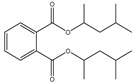	<5	<10
DMEP	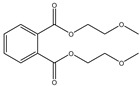	<5	<10
DEEP	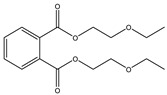	<5	<10
DBEP	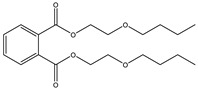	<5	<10

**Table 2:** Detection results of DMP and DBP in baijiu and plastic bottled drinks (*n* = 3).

Samples	Channel	Target	Added Level		
			0	20 μg/L	50 μg/L
Baijiu	B value	DMP	180.5 ± 9.5	144.1 ± 0.2	131.1 ± 0.2
		DBP	176.1 ± 12.8	147.7 ± 0.4	131.4 ± 0.5
	Colorimetric immunoassay *^a^*	DMP	ND	20.1 ± 0.3	48.2 ± 2.1
		DBP	ND	19.5 ± 1.4	48.3 ± 0.9
	ELISA *^b^*	DMP	ND	20.4 ± 0.2	49.6 ± 1.3
		DBP	ND	19.6 ± 1.1	50.4 ± 0.7
Drinks	B value	DMP	179.3 ± 10.2	144.5 ± 0.6	130.9 ± 0.5
		DBP	174.4 ± 11.6	146.3 ± 0.7	130.9 ± 0.4
	Colorimetric immunoassay	DMP	ND	19.6 ± 0.7	48.70 ± 2.5
		DBP	ND	19.9 ± 0.2	49.2 ± 0.9
	ELISA	DMP	ND	20.1 ± 0.4	49.1 ± 1.3
		DBP	ND	20.3 ± 0.7	48.9 ± 0.8

*^a^* *^b^* The unit of value is μg/L.

## Data Availability

The datasets generated for this study are available on request to the corresponding author.

## Supplementary Materials

The following supporting information can be downloaded at: https://www.mdpi.com/article/10.3390/bios13100919/s1, Experimental methods, Figure S1: Standard liquid test areas and sample test areas of the target; Figure S2: The size distribution of the Pt@Au nanozyme; Figure S3: ELISA standard curve for DMP in PBS; Figure S4: ELISA standard curve for DBP in PBS; Table S1: Experimental results of catalytic performance of the Pt@Au nanozymes.
