# Supplementary material for: A Nanozymatic-Mediated Smartphone Colorimetric Sensing Platform for the Detection of Dimethyl Phthalate (DMP) and Dibutyl Phthalate (DBP)
Source: Biosensors (Basel). 2023 Oct 8;13(10):919. doi: 10.3390/bios13100919 (PMC10605576; doi:10.3390/bios13100919)
Supplement: Supplementary file 1 [file biosensors-13-00919-s001.zip › biosensors-2630451-supplementary.pdf]

Supporting information

A Nanozymatic-Mediated Smartphone Colorimetric Sensing Platform for the Detection of Dimethyl Phthalate (DMP) and Dibutyl Phthalate (DBP)

Wenhui Li [1], Xuecheng Zhang [2], Haojie Zhang [2], Cheng Zhang [2], Yingjie Chen [2], Cong Li [3], Yonghong Hu [1], Xiaoping Yu [2], Biao Zhang [2] and Xiaodong Lin [4,*]

1 College of Food and Light Industry, Nanjing Tech University, Nanjing 211816, China; li3781692@njut.edu.cn (W.L.); hyh@njut.edu.cn (Y.H.)
2 Zhejiang Provincial Key Laboratory of Biometrology and Inspection & Quarantine, College of Life Sciences, China Jiliang University, Hangzhou 310018, China; zhangxuecheng@cjlu.edu.cn (X.Z.); zhj20@cjlu.edu.cn (H.Z.); zhangcheng@cjlu.edu.cn (C.Z.); cyj221@cjlu.edu.cn (Y.C.); yxp@cjlu.edu.cn (X.Y.); zb@cjlu.edu.cn (B.Z.)
3 Agriculture and Rural Bureau of Zhuozhou, Zhuozhou 072750, China; lic1995@126.com
4 Zhuhai UM Science & Technology Research Institute, Zhuhai 519000, China
* Correspondence: zumri.xdlin@um.edu.cn

Table S1

Figure S1

Figure S2

Figure S3

Figure S4

Table S1. Experimental results of catalytic performance of Pt@Au nanozyme.

	Tube 1	Tube 2	Tube 3	Tube 4
TMB color development solution (μL)	550.0	550.0	550.0	550.0
Water (μL)	10.0	0	0	0
Diluted Pt@Au (μL)	0	5.0	10.0	20.0
B value	0	22.5±7.4	40.7±5.8	100.5±6.3

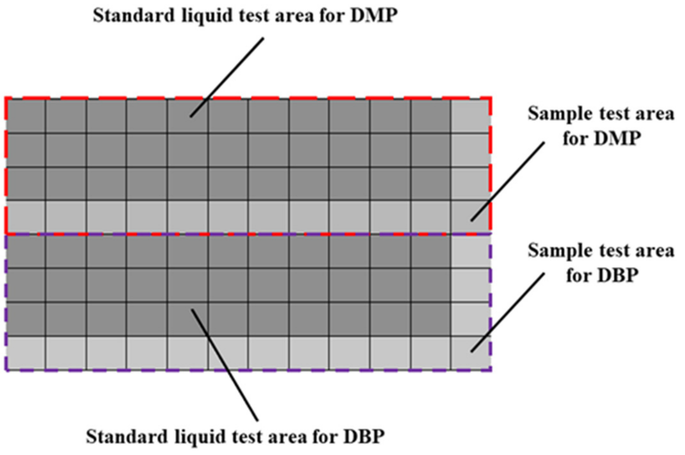

Figure S1. Standard liquid test areas and sample test areas of DMP and DBP in 96 Microporous plates.

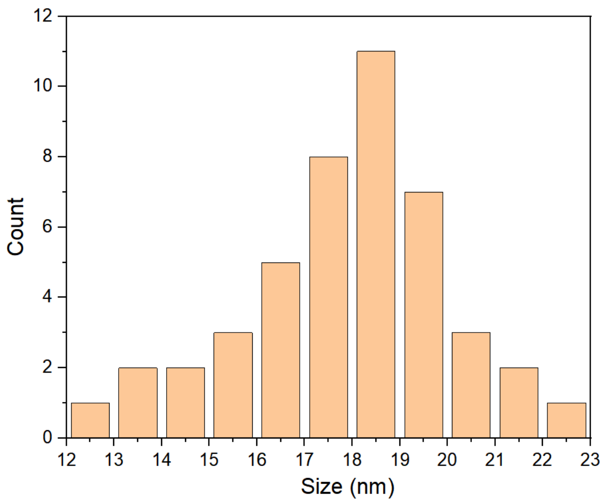

Figure S2. The size distribution of Pt@Au nanozyme.

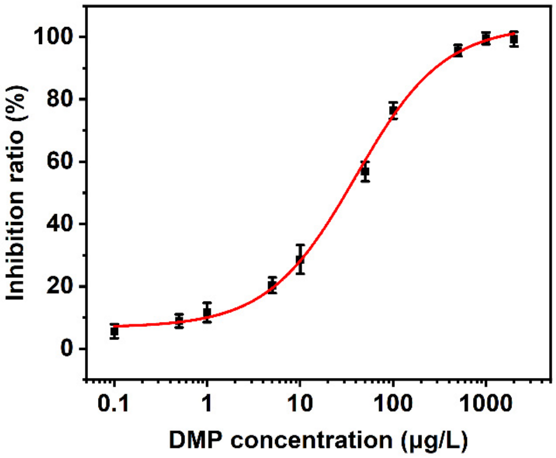

Figure S3. ELISA standard curve for DMP in PBS..

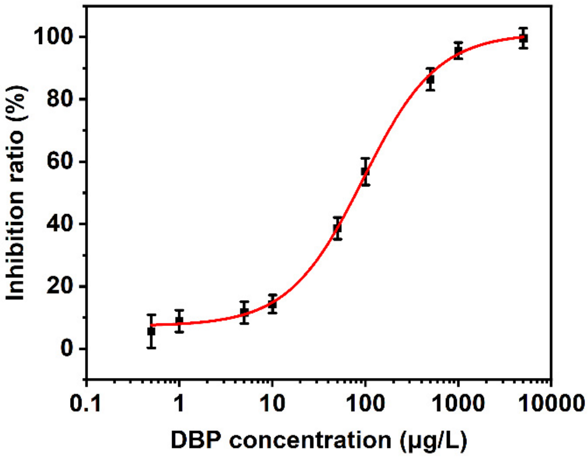

Figure S4. ELISA standard curve for DBP in PBS.
